# ESKAPEE Pathogen Biofilm Control on Surfaces with Probiotic *Lactobacillaceae* and *Bacillus* species

**DOI:** 10.3390/antibiotics12050871

**Published:** 2023-05-08

**Authors:** Claudio Neidhöfer, Kamni Rathore, Marijo Parčina, Martin A. Sieber

**Affiliations:** 1 Institute of Medical Microbiology, Immunology and Parasitology, University Hospital Bonn, Venusberg-Campus 1, 53127 Bonn, Germany; 2 Institute for Functional Gene Analytics, Bonn-Rhein-Sieg University of Applied Sciences, 53757 Sankt Augustin, Germany

**Keywords:** surface sanitization, healthcare-associated infections (HAI), biofilm-related infections, biofilm removal, infection prevention, pathogen control, nosocomial infections, ESKAPEE pathogens, probiotic cleaning, probiotic-based cleaning formulations

## Abstract

Combatting the rapidly growing threat of antimicrobial resistance and reducing prevalence and transmission of ESKAPEE pathogens in healthcare settings requires innovative strategies, one of which is displacing these pathogens using beneficial microorganisms. Our review comprehensively examines the evidence of probiotic bacteria displacing ESKAPEE pathogens, with a focus on inanimate surfaces. A systematic search was conducted using the PubMed and Web of Science databases on 21 December 2021, and 143 studies were identified examining the effects of *Lactobacillaceae* and *Bacillus* spp. cells and products on the growth, colonization, and survival of ESKAPEE pathogens. While the diversity of study methods limits evidence analysis, results presented by narrative synthesis demonstrate that several species have the potential as cells or their products or supernatants to displace nosocomial infection-causing organisms in a variety of in vitro and in vivo settings. Our review aims to aid the development of new promising approaches to control pathogen biofilms in medical settings by informing researchers and policymakers about the potential of probiotics to combat nosocomial infections. More targeted studies are needed to assess safety and efficacy of different probiotic formulations, followed by large-scale studies to assess utility in infection control and medical practice.

## 1. Introduction

Antimicrobial resistance (AMR) is a major global health threat, resulting in millions of deaths each year from antibiotic-resistant infections [1,2]. The most common causes of these infections are a group of bacteria known as ESKAPEE pathogens (*Enterococcus faecium*, *Staphylococcus aureus*, *Klebsiella pneumonia*, *Acinetobacter baumannii*, *Pseudomonas aeruginosa*, *Enterobacter* species, and *Escherichia coli*). As patients with weakened immune systems and antibiotic-induced dysbiosis are at increased risk of developing these infections, hospitals are particularly vulnerable to the spread of ESKAPEE pathogens [3,4]. Reducing the transmission of these infections in hospitals is critical to addressing the overall public health threat posed by AMR [3,5,6].

To reduce the risk of hospital-acquired infections (HAIs) in hospitals and other healthcare facilities, commonly, disinfectants are used. However, the overuse and improper use of disinfectants favor the development and spread of AMR organisms through the selection pressure exerted on microbial populations [7,8,9,10,11]. The reduction in susceptible microorganisms facilitates multiplication and overgrowth of the resistant ones. In addition, the widespread use of disinfectants can also lead to the emergence of cross-resistance [12,13].

In recent years, there has been interest in using probiotic-based cleaning formulations as alternative cleaning agents in hospitals and other healthcare settings [8,10,11,14,15,16,17,18]. Unlike traditional disinfectants, which unselectively eliminate microorganisms, probiotics used in this context are more selective and might only target specific microorganisms by directly eliminating or suppressing growth. This means that they are less likely to lead to the development of AMR strains of bacteria. There is some evidence to suggest that probiotic-based cleaning formulations may be effective at reducing the risk of HAIs in hospitals [8,14,15,16]. However, more research is needed to confirm the effectiveness of probiotics as a standalone cleaning agent in the hospital setting. The effectiveness of probiotics in reducing the risk of HAIs depends on various factors, including the specific strain or strains of bacteria used, the dose and mode of application of the probiotic, and the patient population under study.

In-depth analysis is needed to identify which specific probiotic bacterial strains are most effective in reducing the risk of hospital-acquired infections. While *Lactobacillaceae* and *Bacilli* have demonstrated their potential in some studies, it is crucial to consider the unique properties of different probiotic strains and the specific conditions in which they are used. It is also essential to consider the ways in which probiotic strains may interact with each other, conventional antimicrobials, and the potential for adverse effects in certain patient populations. In this review, we focus on the effect of probiotic *Lactobacillaceae* and *Bacilli* as well as their products in the direct and indirect elimination of ESKAPEE pathogens.

## 2. Results

### 2.1. Species Reported to Suppress ESKAPEE Pathogen Growth

Among *Lactobacillaceae*, *Lactiplantibacillus plantarum* was the most frequently reported species, with 36 instances of growth inhibiting, bactericidal, or anti-biofilm properties against at least one ESKAPEE pathogen [19,20,21,22,23,24,25,26,27,28,29,30,31,32,33,34,35,36,37,38,39,40,41,42,43,44,45,46,47,48,49,50,51,52,53,54]. *Lactobacillus fermentum* [21,22,28,30,31,43,55,56,57,58,59,60,61,62,63,64,65] and *Lacticaseibacillus rhamnosus* [19,21,25,27,31,66,67,68,69,70,71,72,73,74,75,76,77] were both reported 17 times, *Lactobacillus acidophilus* was reported 16 times [21,28,55,67,78,79,80,81,82,83,84,85,86,87,88,89]. The next most common species were *Lactobacillus paracasei* (11 times) [19,20,58,73,74,77,90,91,92,93,94], *Lactobacillus casei* (9 times) [88,95,96,97,98,99,100,101,102], *Limosilactobacillus reuteri* (6 times) [19,103,104,105,106,107], *Levilactobacillus brevis* (6 times) [19,25,29,108,109,110], *Lactobacillus salivarius* [50,111,112,113] and *Lactobacillus helveticus* [40,102,114,115] (4 times), *Lactobacillus delbrueckii* [19,116,117] and *Lactobacillus crispatus* [65,118,119] (3 times), *Lactobacillus pentosus* [26,120], *Lactobacillus gasseri* [119,121], and *Lactobacillus curvatus* [51,102] (2 times). *Lactobacillus agilis* [122], *Lactobacillus caucasicus* [19], *Lactobacillus gallinarum* [31], *Lactobacillus gastricus* [90], *Lactobacillus johnsonii* [24], *Lactobacillus kunkeei* [123], *Lactobacillus murinus* [124], *Latilactobacillus sakei* [102], *Lactobacillus vaginalis* [106] and *Lacticaseibacillus zeae* [58] were each only reported once. Among *Bacilli*, *Bacillus subtilis* was the most frequently reported species (five times) [125,126,127,128,129], followed by *Bacillus velezensis* (two times) [130,131]. *Bacillus thuringiensis* [46], *Bacillus amyloliquefaciens* [126], *Bacillus cereus* [132] and *Bacillus pumilus* [133] were each reported once.

### 2.2. Origin of the Isolates

Among cases where information on the origin of the isolates was provided, probiotic bacteria were most frequently isolated from more and less fermented food (22 times) [20,22,26,41,43,44,45,48,49,74,95,102,107,109,110,128,134,135,136,137,138,139] and dairy products (11 times) [24,31,40,57,58,69,79,86,90,97,108,117]. Some were of human origin; these included bacteria of vaginal origin (six times) [64,65,72,93,119,140], such isolated from infant GI-tracts (six times) [24,42,62,77,94], and bacteria from the oral cavity (three times) [56,58,78], intestine (two times) [59,141] and one from breastmilk [75]. Others were of animal origin; these included three of bovine origin [25,29,33], two isolated from pigs [122,142], two from bees [123,131], and one from poultry [106], a dog [124], a bullfrog [51] and camel milk [23], respectively. Two *Bacillus* isolates were of marine origin [127,133]. Information on the origin of the remaining isolates was either not available or they were derived from probiotic bacteria-based products or microbiological strain collections.

### 2.3. Nature of Conducted Experiments

The vast majority of studies were based on in vitro experiments (132 times) in which the effects of probiotic bacteria against pathogens were mainly evaluated by agar spot tests, agar well tests, co-culture in liquid media and on the surface of human cell lines. The latter included Caco-2 [44,50,89,103], HT29 [24,89,101], HeLa 229 [65,83], vaginal epithelial cells [71], and uroepithelial cells [143]. Some of the in vitro experiments tested the anti-biofilm properties of the probiotic bacteria on inert surfaces [81,93,125], most importantly on metals such as stainless steel [120] or titanium [28]. Other surfaces included those made of silicon [32], ceramics, and linoleum [144]. Six studies were based exclusively on in vivo experiments. These included three rat disease models, two of which were for wounds [53,54] and one was for surgical implants [55], two murine disease models [145], one of which also included a bovine disease model [130], one rabbit model knee implant infection [76] and one bee model [123]. Three studies were based on combined in vitro and in vivo experiments and exclusively included murine disease models, two intestinal colonization models [36,52] and one urinary tract infection model [113]. In one of the latter, the anti-pathogenic properties demonstrated in vitro could not be replicated in vivo [52].

### 2.4. Suppression of ESKAPEE Pathogens

The ESKAPEE pathogens that were most frequently reported to be displaced by the probiotic bacteria were by far *Staphylococcus aureus* (77 times) and *Escherichia coli* (73 times). The next most common ESKAPEE pathogen to be described as having been displaced was *Pseudomonas aeruginosa* (30 times). These three were also by far the most frequently tested ESKAPEE pathogens. Table 1 displays the number of times each probiotic species was reported to displace these three pathogens. Less frequently tested and displaced were *Klebsiella pneumoniae* (nine times) [26,43,52,56,65,79,132,136,146], *Enterococci* (five times) [85,103,107,141,147], *Enterobacter* species (two times) [26,61] and *Acinetobacter baumannii* (once) [68]. Of the *Enterococcus* isolates, four were *E. faecalis* and one *E. faecium*. Nine reports described pathogen inhibition to be caused by direct competition between probiotic and pathogenic bacterial cells [24,29,32,40,102,108,135,143,148]. Nineteen studies pinned the bactericidal properties down on one or more specific cell products [46,49,51,56,57,59,73,76,99,100,117,125,127,129,133,136,149,150,151]. Eleven studies ascribed the suppression of pathogens to be attributable to both, direct competition and cell products [27,55,61,64,72,75,77,104,116,126,152]. Five studies linked attributed bactericidal activity exclusively to lactic acid and pH reduction [98,119,137,138,153]. The remaining 52 studies that identified the inhibition mechanism narrowed it down to be the cell-free supernatant (CFS) or exclusively investigated the CFS of probiotic bacteria. Nigatu and Gashe (1994) reported inhibitory effects to be independent of pH [136], Kang et al. (2017) discovered that pH neutralized CFS was still efficient in killing pathogenic bacteria as long as it was not heat inactivated or proteinase K treated [111]. Jeyanathan et al. (2021) described whole cell cultures to be ineffective in preventing *P. aeruginosa* growth, while CFS significantly reduced adherence and viability [28].

Twenty-four studies described tested probiotic bacteria as having significant anti-biofilm properties. These biofilms were of *S. aureus* (14 reports) [22,24,25,42,59,68,81,88,95,107,108,114,130,154], *E. coli* (9 reports) [24,26,60,68,70,95,101,152,154], *P. aeruginosa* (7 reports) [26,42,59,113,123,153,155], *K. pneumoniae* (2 reports) [26,146], *E. faecalis* (1 report) [107], *Enterobacter* (1 report) [26] and *A. baumannii* (1 report) [68] isolates. Biofilm impairment was generally by co-aggregation [24,81,108], by reducing pathogen adhesion [42,60,68,101,114], or by disrupting cell metabolism or interfering with quorum sensing [46,70]. Chappell and Nair (2020) determined *P. aeruginosa* inhibition to be dependent on pH reduction [153]. Koohestani et al. (2018) discovered *Lact. acidophilus* CFS to better remove *S. aureus* biofilms than *Lact. casei* CFS [88]. Five authors reported the probiotic bacteria capable of replacing the pathogens’ biofilms with their own, which subsequently prevented recolonization by the pathogenic species [25,60,68,123,152]. Several additional studies reported the ability of pathogens to adhere and survive on probiotic-covered surfaces or such covered with probiotic-products to be significantly reduced [27,30,32,37,55,102,125,135,144,156]. Kheiri et al. (2020) discovered *Lactobacillus* CFSs, even in 1:16 dilutions, to have superior biofilm-inhibiting and -killing properties than supra-MIC levels of several tested antibiotics [146].

Among studies evaluating the activity of probiotic bacteria or their products against *S. aureus*, eight included methicillin-resistant *S. aureus* (MRSA) strains [21,53,75,121,126,127,131,151]. Kalayci Yüksek et al. (2021) detected low protective effect of *Lact. acidophilus*, *Lact. plantarum*, *Lact. fermentum* and *Lact. rhamnosus* on MRSA [21]; Sürmeli et al. (2019) detected no therapeutic effect but good protective effect of *Lact. plantarum* in preventing MRSA colonization when applied before MRSA on wounds [53]. Algburi et al. (2021) determined CFS of *B. subtilis* and *B. amyloliquefaciens* to inhibit both MRSA and methicillin-susceptible *S. aureus* (MSSA) [126]. Liu et al. (2020) reported also *Lact. rhamnosus* to inhibit both MSSA and MRSA in vitro and in the murine model [75]. Kalpana et al. (2012) described the products of *B. subtilis* to inhibit MRSA but not *P. aeruginosa* [127]. Klimko et al. (2020) discovered *Lact. acidophilus* to be the strongest *S. aureus* inhibitor among nine tested probiotic isolates [19].

Two studies evaluating the activity of probiotic bacteria or their products against *E. coli* included enterohemorrhagic (EHEC) strains [96,135], two included enteropathogenic EPEC strains [36,89], and one contained an extended-spectrum beta-lactamase-encoding *E. coli* [79]. All reported the tested *Lactobacillales* to displace the pathogens. Six studies included not only ESKAPEE pathogens but also other *Enterobacterales* that frequently cause HAIs, such as *Proteus mirabilis* [61,65,157] and *Proteus vulgaris* [51], *Klebsiella oxytoca* [61] and *Klebsiella aerogenes* [129], *Citrobacter freundii* [51,61], and *Serratia marcescens* [140] to be inhibited by probiotic bacteria. Scillato et al. (2021) reported *Lact. fermentum* and *Lact. crispatus* to displace *P. mirabilis* and even KPC carbapenemase-encoding *K. pneumoniae*, but not *E. faecalis*, vancomycin-resistant *E. faecium*, or *Candida albicans* [65]. In the meantime, Strus et al. (2020) only discovered a few *lactobacilli* able to inhibit *E. faecalis* [158]. De Souza Freitas et al. (2020) determined *B. subtilis* CFS to also inhibit less frequent nonfermenting HAI-causing pathogens *Achromobacter xylosoxidans*, *Alcaligenes faecalis*, and *Pseudomonas alcaligenes* [129]. Turkova et al. (2013) reported *Lact. acidophilus*, *Lact. gasseri*, and *Lact. helveticus* to inhibit *E. coli*, *E. faecalis* and even *Clostridioides difficile*, while *S. aureus* was not inhibited by *Lact. Helveticus* [147]. One study reported some *Lactobacillales* to enhance *E. coli* growth [33].

## 3. Discussion

The protective effects of colonizing the human body [159,160,161,162,163,164,165] or food [159,166,167] with probiotics have been well studied. However, applying this concept to infection vector-transmitting surfaces such as those found in hospitals through protective probiotic films is a novel approach that challenges traditional infection prevention strategies centered on maintaining a sterile hospital environment. Probiotic cleaning formulations show promise as a potential solution to the proliferation of antimicrobial resistance, particularly in healthcare settings [8,10,11,14,15,16,17]. These products, which contain live microorganisms beneficial to the cleaned environment, may restore microbial balance and reduce selective pressure driving drug-resistant bacteria. They may also have a more favorable environmental profile due to their use of natural, biodegradable ingredients.

Our review of the efficacy of probiotics in reducing the presence of ESKAPEE pathogens identified numerous studies that analyzed the bactericidal properties of probiotic strains against *E. coli* and *S. aureus*, which are well-known causes of gastroenteritis [168] particularly relevant to the use of probiotics in the gastrointestinal tract. The probiotic isolates examined in these studies were primarily *Lactobacilli*, aligning with the focus on using probiotics in the gut. In addition, most studies simulated conditions present in the gastrointestinal tract. *P. aeruginosa*, a microorganism known to cause food spoilage [169], was also frequently examined for its susceptibility to probiotics. However, there were very few studies that investigated the effects of probiotics against the majority of other ESKAPEE pathogens.

It is important to note that the greater number of studies demonstrating the bactericidal effects of *Lactobacillales* does not necessarily indicate superior performance in suppressing ESKAPEE pathogens compared to *Bacillus* species. Rather, evaluating probiotics for use on hospital surfaces requires a more nuanced analysis that considers factors such as survivability, biofilm formation, and bactericidal activity at room temperature and under nutrient-poor conditions, which may instead make *Bacillus* species more attractive candidates. It is also crucial to recognize that significant strain-level variations within the same species can impact the desired properties [170,171].

Despite the generally recognized safety of probiotics and the fact that most were derived from benign edible sources some of which are even consumed in large quantities, the use of these microorganisms in hospitals warrants further investigation to ensure their safety, particularly in dysbiotic, immunocompromised, leukopenic or even agranulocytic patients [172,173,174,175], as these are patient groups that could particularly benefit from such innovative solutions [176,177,178,179,180,181,182,183,184]. It is desirable for these microorganisms, which may be transferred from the environment to the patient, to retain their protective effects in the patient as well.

Conducting more targeted studies on hospital or similar surfaces and the specific pathogens to be displaced provide valuable insights and aid in the development of new and promising approaches for preventing or eradicating bacterial biofilms in medical settings [185,186,187]. Comparison of different probiotic-based cleansing formulations should also be considered, as the efficacy or inefficacy of one product may not necessarily apply to other products with different strains.

## 4. Materials and Methods

A systematic search was conducted using the PubMed and Web of Science databases. The search was performed on 21 December 2021. The search string used was the following:

“(Probiotic-Based Cleaning OR Antagonistic Activit* OR Inhibition OR Disinfectant OR Anti-infective OR Biofilm removal OR cleaning solution* OR infection control OR antibiofilm OR biosurfactants OR Sanitation OR Surfactants OR Cleaning OR Bacteriostatic OR Antimicrobial OR Microbial based cleaning OR Antagonistic activity OR Lantibiotics OR bacteriocins OR antagonistic activity) AND (biofilm OR Surface OR medical device) AND (*Staphylococcus aureus* OR *Klebsiella pneumoniae* OR *Acinetobacter baumannii* OR *Pseudomonas aeruginosa* OR *E. coli* OR Enterobac* OR Nonfermen* OR MRSA OR CRE OR MDR) AND (Lactobacill* OR Probiotics OR *Bacillus* OR Clostridi* OR Bacteroi*) NOT “Review” [pt]”.

For the assessment of search results, results were imported into CITAVI Database. Two reviewers independently screened the titles and abstracts of the identified studies for eligibility. Studies were eligible for inclusion if they examined the effects of *Lactobacillus*, *Lacticaseibacillus*, *Lactiplantibacillus*, *Limosilactobacillus*, *Latilactobacillus* or *Bacillus* spp. or their products/supernatants on one or more ESKAPEE pathogens. ESKAPEE pathogens were defined as *Enterococcus faecalis*, *Enterococcus faecium*, *Staphylococcus aureus*, *Klebsiella pneumoniae*, *Acinetobacter baumannii*, *Pseudomonas aeruginosa*, *Enterobacter* species, and *Escherichia coli*. Full-text articles were then obtained for all potentially eligible studies and assessed for inclusion based on the predefined inclusion and exclusion criteria. Any discrepancies between the reviewers were resolved through discussion and consensus.

**Figure 1:**
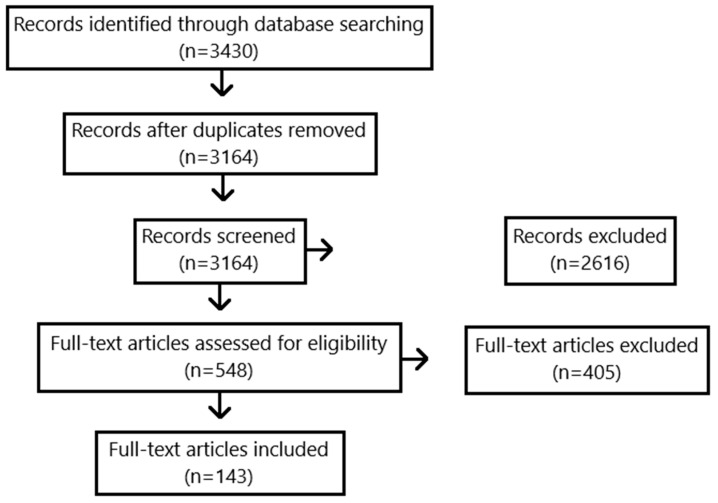
Study flow diagram.

Overall, a total of 143 studies were included in the review (see Figure 1). The studies were conducted in a variety of settings and involved a range of bacterial strains and ESKAPEE pathogens. The experiments included both in vitro and in vivo studies, and a variety of methods were used to assess the effects of the probiotic bacteria on the growth, colonization, and survival of the pathogens.

Data were extracted from the included studies using a standardized data extraction form. The following data were extracted: examined bacteria, ESKAPEE pathogens they were tested against, environment tested in, type of experiment, and, if applicable, additional relevant information. The extracted data were synthesized and analyzed using a narrative synthesis approach. The results were organized by bacteria and pathogens tested and summarized in a tabular format.

Our review aimed to provide a comprehensive overview of the available evidence on the effects of *Lactobacilli* and *Bacilli* or their products against ESKAPEE pathogens. By synthesizing and analysing the data from the included studies, we aimed to evaluate the potential of these probiotic bacteria as means to combat infections caused by these pathogens.

## 5. Conclusions

The evidence collected from the multitude of scientific studies shows that many *Lactobacillaceae* and *Bacillus* species are able to suppress the proliferation of ESKAPEE pathogens. However, the experimental conditions under which these studies were conducted do not allow conclusive assessments of the efficacy of the species in protecting hospital surfaces, which could contribute to the development of new promising approaches to prevent or eradicate bacterial biofilms in medical settings. More targeted studies on hospital surfaces and pathogens to be displaced are needed to understand the potential of each species and strain in more detail, along with their respective safety profiles. Once a safe and effective formulation is identified, conducting large-scale studies seems imperative, given the potential they carry in tackling several issues infection control and medical practice in general are currently facing.

## Figures and Tables

**Table 1:** List of probiotic bacteria, and number of cases in which antibacterial properties were attributed to them against one or more of the three most frequently tested ESKAPEE pathogens.

	*S. aureus*	*E. coli*	*P. aeruginosa*
*Lactiplantibacillus plantarum*	23	16	12
*Lactobacilus fermentum*	7	5	7
*Lacticaseibacillus rhamnosus*	8	9	2
*Lactobacillus acidophilus*	8	7	5
*Lactobacillus paracasei*	5	7	3
*Lactobacillus casei*	5	6	-
*Limosilactobacillus reuteri*	4	3	1
*Levilactobacillus brevis*	5	4	1
*Lactobacillus salivarius*	3	-	1
*Lactobacillus helveticus*	2	3	-
*Lactobacillus delbrueckii*	1	2	-
*Lactobacillus crispatus*	1	2	2
*Lactobacillus pentosus*	1	2	1
*Lactobacillus curvatus*	1	1	1
*Lactobacillus caucasicus*	1	1	1
Other *Lactobacillaceae*	20	25	6
*Bacillus subtilis*	3	2	1
*Bacillus cereus*	1	1	1
Other *Bacillus* spp.	4	2	1

## Data Availability

Not applicable.
